# Bridging the Gap: Understanding Patient and Clinician Preferences When Designing Preoperative Education Programs

**DOI:** 10.1111/jep.14259

**Published:** 2024-12-11

**Authors:** Rochelle Furtado, Joy C. MacDermid, Christina Ziebart, Dianne Bryant, Kenneth J. Faber

**Affiliations:** ^1^ Department of Rehabilitation Sciences Faculty of Health Science, Western University London Ontario Canada; ^2^ Collaborative Program in Musculoskeletal Health Research Bone and Joint Institute, Western University London Ontario Canada; ^3^ School of Physical Therapy, Faculty of Health Science Western University London Ontario Canada; ^4^ Department of Surgery Western University London Ontario Canada; ^5^ Roth McFarlane Hand and Upper Limb Centre, St. Joseph's Hospital London Ontario Canada

## Abstract

**Background:**

Traditionally, health information has been created from the perspective of the providers with minimum patient consultation, hindering engagement and adherence. The rate of shoulder replacements has increased over the past decade, is associated with shorter hospital stays, and patients are relying on education to be able to participate in shared decision‐making. Therefore, to ensure creation of accessible education programs for shoulder replacement procedures, we explored patient and clinician preferences regarding content and device choices for a preoperative shoulder replacement education program.

**Methods:**

This study used an interpretive descriptive qualitative approach to understand patient and clinician preferences. We included a subset of patient and healthcare provider perspectives, from those who had previously completed our quantitative survey. Interviews were conducted in English by one researcher. Analysis was conducted through a descriptive thematic analysis with open coding.

**Results:**

A total of 10 patients and 9 healthcare providers were interviewed. Findings were categorized into four main themes described the process of creating patient education material (1) methods of accessing information, (2) deciding on educational content, (3) deciding on device use, and the last theme of factors affecting engagement can be further divided as (4‐1) promotors of engagement and (4‐2) barriers of engagement.

**Conclusions:**

A multimodal program of a website with videos and a written booklet, that covers basic information regarding the surgery, timelines for recovery, sling use, use of therapeutic devices/aids post‐surgery, patient expectations to improve surgery satisfaction, postoperative restrictions, pain management, rehabilitation and home supports is desired by both patients and clinicians.

## 1 Background

Despite preoperative education programs showing positive change in postoperative outcomes such as increased patient knowledge and behavior change in cardiac surgeries and reduced anxiety in cancer surgeries [1], the uptake of these programs in orthopedics is lagging [2]. Poor uptake of preoperative programs has been attributed to the mixed evidence on whether these programs can truly affect postoperative outcomes [3, 4, 5]. As shown in our prior work [2], many studies of preoperative education programs have reported an inconclusive effect on postoperative outcomes such as lowering postoperative pain or decreasing length of hospital stay [3, 4, 5]. However, these studies have been designed without attention to treatment fidelity, adherence and using health literacy prinicples [2]. Without designing rigorous education programs for patients and their caregivers to better understand the surgery and recovery pathways, we do not equip patients with knowledge to better manage their own recovery pathway [6].

Designing an engaging preoperative education program requires a theoretical framework, clinical expertise, and the knowledge of patient preferences [2, 6]. However, patient preferences during the development of these programs are rarely reported within orthopedic literature. In 2017, a study by Kennedy et al. explored patients' needs for a hip or knee replacement education application [7]. Their findings from patient interviews strengthened the development of their mobile education application because they identified the lack of sufficient pain management education for post‐surgery. Traditionally, patient education programs have been created from the perspective of the providers and neglected patients’ perspectives which potentially hinders patient engagement and adherence with these programs [7, 8].

Using a design process that entails both clinician and patient perspectives when creating a program is commonly referred to as patient Codesign. Codesign is part of a process to (1) engage with people; (2) capture patient, family and staff experiences; (3) organize the learning from captured experiences to create new understanding and insight from the perspective of the care journey and emotional journey; (4) come together in partnership to review the learning, have ideas, plan and implement the ideas; (5) review and reassess the new implemented ideas [9]. This method uses the experiences that patients and the public have when they receive healthcare services to change care and transform services.

Recently, a systematic review evaluating the development process of mobile health applications within rheumatic and musculoskeletal diseases emphasized that the absence of involving stakeholders could lead to fallible conclusions about patient needs and preferences [10]. Not including patient partners leads to the creation of programs that do not accommodate patient needs, health literacy levels, and learning styles. The overestimation of health literacy and education is associated with difficulties in understanding health information [11]. These assumptions tend to limit the usability and design of patient education programs. The rate of shoulder replacement surgery has increased [12], is associated with shorter stays at hospital, and patients are relying on more education to be able to participate in shared decision‐making. Therefore, to ensure researchers can create accessible and engaging education programs for shoulder replacement procedures, we explored patient and clinician preferences regarding content and device choices for a preoperative shoulder replacement educational program.

## 2 Methods

### 2.1 Study Design

This study used an interpretive descriptive qualitative approach to understand patient and clinician preferences for creating a preoperative patient education program for those undergoing a shoulder replacement. Interpretive description is a method that generates knowledge that is relevant for the clinical context of a variety of applied health disciplines. As used in previous studies, this methodology aims to generate practise‐relevant findings that are useful for program developement [13, 14, 15].

### 2.2 Setting and Sample

Interviews were conducted online using videoconference software (Zoom, San Jose CA), in a small private room within a dedicated clinical research laboratory. Participants were recruited from a previous quantitative survey where participants indicated their interest in completing a qualitative interview to further understand their preferences [16]. Patients were eligible to complete the anonymized survey if they were on a waiting list for a shoulder replacement within a Canadian hospital. Eligible patients had to be over the age of 18 years and able to read and write in English. Clinicians were also eligible to complete the survey if they were over the age of 18 years, practiced and/or had experience with upper extremity surgical patients, and able to read and write in English.

Through purposeful sampling, we included a subset of perspectives who had previously completed our quantitative survey and indicated they were interested in completing a qualitative interview. Results from the quantitative survey have been previously published [16]. Participants were either healthcare providers or patients on the waiting list for surgery. Healthcare providers were clinicians involved in the perioperative care of patients awaiting shoulder replacement and had experience with patient education. We aimed to include the participation of both men and women of varying age groups and ethnicities. Recruitment for interviews stopped when saturation of the responses in both groups was achieved. The study protocol was reviewed and approved by Lawson Healthcare and the Western Research Ethics Board (WREB).

### 2.3 Data Collection

Participants provided written informed consent before the interview. Interviews were conducted in English by one researcher (RF) and lasted on average 35 min. RF identifies as a woman, who was a PhD student and physical therapist at the time, she had 5 years of qualitative experience. Pilot interviews (*n* = 5) were conducted with other researchers before official data collection, to ensure accuracy of the interview questions and minimal bias during the interview process. All interviews were recorded using the videoconference software and transcribed verbatim.

The interview guide was informed by previously published work and multiple discussions with the research team. Interviews focused on responses we obtained from our previous survey on patient education. For patients, questions began with open, broader experiences about access to education, their needs, questions they had about surgery and their level of comfort with various platforms for receiving education. Whereas for clinician participants, questions focused on their experiences with providing education, information they wanted patients to know and their thoughts about various platforms for delivering education. All questions were meant to be exploratory and relied on probes to allow for perceptions and experiences to emerge during the interview.

### 2.4 Analysis

Descriptive statistics were collected for participants. The audio recordings from the Zoom interviews were analyzed by members of the research team (RF and CZ). Analysis was conducted through a descriptive thematic analysis that consisted of open coding. This allowed the transcripts to be separated into fragments for extraction of relevant themes. Next, themes were categorized and co‐created from the responses to each question. Findings were summarized with quotes and diagrams as needed. Final themes were presented to all team members to ensure multiple perspectives were accounted for and knowledge was co‐created within the team.

## 3 Results

A total of 19 participants were interviewed on their preferences for preoperative education programs: patients (*n* = 10) and healthcare providers (*n* = 9). Clinicians interviewed included: surgeons (*n* = 3), physical therapists (*n* = 4) and occupational therapists (*n* = 2). Tables 1 and 2 detail demographic information about the participants.

**Table 1:** Demographic information for clinicians.

Participant ID	Profession	Gender	Years of clinical experience
Clinician 1	Physiotherapist	Male	5 years
Clinician 2	Orthopedic Surgeon	Male	5+ years
Clinician 3	Orthopedic Surgeon	Male	5+ years
Clinician 4	Occupational Therapist	Female	15+ years
Clinician 5	Occupational Therapist	Male	5 years
Clinician 6	Physiotherapist	Male	15+ years
Clinician 7	Physiotherapist	Female	5 years
Clinician 8	Physiotherapist	Female	5+ years
Clinician 9	Orthopedic Surgeon	Male	5+ years

**Table 2:** Demographic information for patients.

Participant ID	Profession	Gender	Marital status	Caregiver after surgery?
Patient 1	Retired	Female	Married	Yes – Spouse
Patient 2	Retired	Male	Married	Yes – Spouse
Patient 3	Retired	Female	Married	Yes – Spouse
Patient 4	Retired	Female	Married	Yes – Spouse
Patient 5	Retired	Female	Single	No
Patient 6	Retired	Female	Married	Yes – Spouse
Patient 7	Retired	Female	Single	No
Patient 8	Retired	Male	Married	Yes – Spouse
Patient 9	Retired	Female	Widow	Yes – Adult child
Patient 10	Retired	Female	Widow	Yes – Adult child

**Figure 1:**
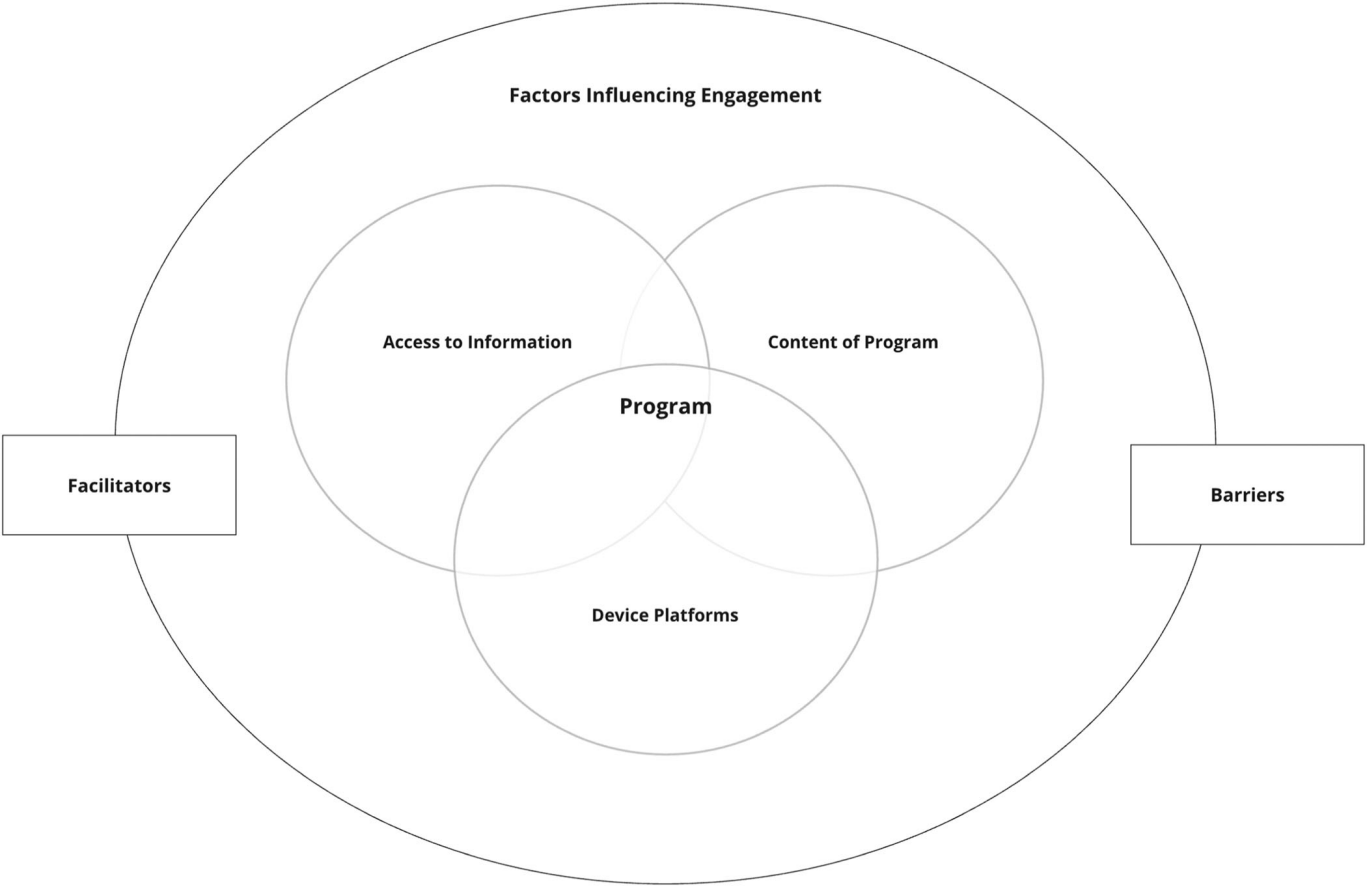
Pictorial figure summarizing the emerging themes and how they intersect when creating a program.

We organized our findings into 4 main themes that best described the process of creating patient education material (1) methods of accessing information, (2) educational content, (3) methods for content delivery device use, and (4) factors affecting engagement were further divided into (4‐1) engagement facilitators and (4‐2) engagement barriers. The themes were summarized into a figure representing the components of creating preoperative patient education materials (Figure 1).

### 3.1 Theme 1: Methods of Accessing Information

A common theme that emerged from the interviews was the concept of accessing healthcare information. When patients described methods of how they commonly access healthcare information, responses included the use of websites like Google, relying on their healthcare provider, or relying on experiences of other patients who have had a similar surgery emerged.

Oh, yeah, my husband and I both went on the computer together, we watched a video for the actual surgery, just you know, we felt it would be good for me to see what was going to happen and hopefully help me deal with what needs to be done after and stuff. And then my brother has had a shoulder surgery, not a replacement …but like having him as a resource as well is nice.

(Patient 1)

No, I think Dr. XXX was very good, and he was good at explaining everything and the recovery time and all of that. So yeah, I felt totally fine in getting information.

(Patient 3)

The majority of clinicians preferred that patients access health information by direct contact in a clinical setting or through online resources that allow patients to complete their own research.

Yeah, I think it's important for us as physicians to be able to educate a patient on their pathology and their diagnosis…You know, it's not uncommon for a lot of patients to actually say oh I just watched this video on YouTube to see what you're going to do.

(Clinician 3)

In summary, the findings in this theme suggest that participants will initially rely on health professionals for information and perform an internet search to supplement the received information, and finally, follow up with a trusted patient who has already experienced shoulder replacement surgery.

### 3.2 Theme 2: Deciding on Educational Content

The content or topics that felt relevant for an education program were the next themes that emerged from the interviews. Common topics included the length of recovery, what happens on the day of surgery, sling use, timelines for follow‐up visits etc. The use of therapeutic devices/aids following surgery was a major concern, especially among patients with no social support at home after surgery.

But, I'm just getting to know for myself like what kind of aids do I need at home to help me because I do live alone, maybe in the first week I'll have somebody with me, but otherwise I don't know what I'll do.

(Patient 5)

Additionally, many patients wanted information about the rehabilitation that would be available after surgery and how it would affect their recovery.

But if I get the operation, I know I'm done doing stuff for a while and that's totally reasonable too. But well, I don't know what or where else to go. Where else do I need to go to get that physio? Who recommends me to go there and what do I?

(Patient 2)

When exploring clinicians' perspectives, surgeons emphasized the importance of covering information about risks, benefits, patient expectations and recovery pathways.

It's our role to be able to describe the risks and benefits first and foremost to make an informed consent, but then to also allow them to have a trajectory of where they should be in, you know, 6 weeks or 3 months, how long they should expect to have recovery and be in the recovery phase for… and then what their expectations to be.

(Clinician 3)

When exploring physiotherapists' preferences on education content, they identified that their role was to provide patients with topics that may not have been covered by the surgeon previously, especially on the role of postoperative rehabilitation.

And I think PT at least in the role that I've played with it within a day surgery unit has to do a little bit with kind of filling the gap. We can provide education regarding postoperative swelling and pain management, as well as immediate postoperative range of motion restrictions or precautions… things like driving or, or grocery shopping or anything like that, especially the driving component.

(Clinician 1)

A priority for occupational therapists was to include educational content surrounding home supports and the use of caregivers.

If they know before time they need to, then they can make the accommodations and then afterwards, you're not searching for someone or thing, they already have all those in place when they were ready to mobilize.

(Patient 2)

In summary, the findings in this theme suggest that participants want a program that will cover basic information regarding the surgery (why am I having this, how is this performed, risks/benefits, what happens on the surgery day), timelines for recovery, immobilization/sling use, use of therapeutic devices/aids post‐surgery, patient expectations to improve surgery satisfaction, postoperative restrictions, pain management, rehabilitation after surgery, home supports and the use of caregivers.

### 3.3 Theme 3: Deciding on the Platform

The most suitable platform for providing educational content was the next theme that emerged from the interviews. Between both groups of participants, similar findings resulted in either a website with videos, a booklet or both a website plus a booklet as a multimodal approach.

yeah, reading a booklet for myself would be great then if I have questions I can talk to my husband or he would come and say okay, let's look it up online…and have a look and see what's happening. And so, if it's in a booklet…it will help me more than a computer.

(Patient 5)

But I think a website's pretty good. And you can even have a link to an app from the website, which might be a way to have it as a multimodal…but some people just function better with paper too!

(Clinician)

In summary, the findings in this theme suggest that participants want a platform that allows for a multimodal option of using a website with videos and a booklet to supplement the information from the website.

### 3.4 Theme 4: Factors Affecting Participation

The last theme that emerged from the interviews was on factors that influenced engagement. However, this theme can be further broken into two separate themes facilitators (4‐1) and barriers (4‐2).

### 3.5 Subtheme 4‐1: Facilitators

Suggestions to facilitate participation and adherence to an education program that were identified during the interviews included accessibility, design, and engagement of the program. Some participants described how they lived far away from the hospital and having access to information that could be shared with their caregiver without coming into clinic would increase the accessibility and engagement of this program.

Interviewer: So you mentioned that you're from xxx, which is a bit of a drive to come down to xxx… so it sounds like distance is a factor, and I guess if an education program, would have to happen for you, if it would probably be best to be something more virtual or on Internet, so that you could kind of watch on your own time, rather than like have to come into in person to a class?

Participant: oh yes certainly, it would lessen me from having to commute many times…share this with my family!

(Patient 1)

Some patients described the benefits of having access to educational material through multiple modalities such as online material and/or a written booklet that can be reviewed multiple times to improve information retention. This method of instruction would also ensure that the information provided to patients and their families was streamlined and uniform.

If I get to fall back on something…oh gee, I can't remember what the doctor said, or did he say this? or did I say the right thing? And then, if I look at something printed, you know, either online or both, then I could maybe clear my thoughts more.

(Patient 2)

Another facilitator towards clinician engagement with using a preoperative program arose from the limited time clinicians have with a patient during an in‐person appointment. Access to educational material can supplement information they had received from their clinician, fill knowledge gaps that were not discussed during the appointment and can facilitate questions for the following visit. Additionally, standardized information that patients are receiving between multiple healthcare providers.

That's the other thing about this center, in particular, is that it sees a lot of patients that have either are already been seen, and perhaps managed to a non‐ operative extent…and so they're seeing them at their essentially last resort. To be able to say well you need to surgery and a lot of times, you know, I'd say the consultants here are booking their shoulder surgeries almost at the first time they're meeting patients. So, you know, in that sense, they're probably spending 10 min with them at max, and so I don't know if you necessarily go through the whole gamut that we talked about, but I think it's, you know, there are certain portions of it that that are a must and certain portions of it that sometimes get crossed over and sometimes they don't. It really depends on who's giving that information … so it's very variable.

(Clinician 3)

### 3.6 Subtheme 4‐2: Barriers

Barriers to the design or implementation of this program and solutions to mitigate these barriers were the last themes that emerged from the interviews. The importance of using patient‐friendly language/lay terminology when describing medical concepts was a common suggestion from many patients.

I think online videos and things like that could be helpful. It's just important to understand some of the technical pieces of it [surgery], as best as one can and by using simple language. So yeah, that would always be helpful. I mean, having more information is always good for patients.

(Patient 1)

A related concept is the importance of using subtitles or captions to help participants who may have visual/hearing impairments or those who may need subtitles in their native language as the videos would be created in English.

The other thing is to be mindful of accessibility, so I was mindful to have it where it was like a closed caption thing …and making sure just like the audio and visual aligned, because while my patient group wasn't super old, but I think it is important to consider accessibility.

(Clinician 7)

Another barrier that was discussed by a few clinicians was on how to make this education program engaging, but also individualized enough for participants to fully engage.

I think you guys have a tricky task there because engagement is going to be based a lot on the individual and what they want to get out of it. If there was a perfect world, and we could do everything individually one by one, which is probably not feasible to build, I think it's important to focus on the person's goals and then take them through step by step on how we're going to achieve those goals.

(Clinician 6)

A strategy to help overcome these barriers included providing standard information for all participants, followed by opportunities to personalize certain components of the program to meet a specific learning need. For example, participants can navigate through various components of the program and select the sections that meet specific learning needs, and they can select specific stages of pre‐rehab exercise videos based on their current level of activity and pain.

I think the most important thing is probably just giving them a variety of different ways to access the information that they're comfortable with. I always like to use the Windows analogy, where there is 30 different ways to get to your windows computer to any different point and if you watch someone work through that process, it doesn't matter if they click start or if they search or if they use keystrokes of if they use the mouse, they're still going to get to where they need to get to. So not having them needing to do this or needing to that, but having multiple approaches, so they can find the information they want.

(Clinician 6)

Patients were also concerned that information overload could inadvertently create anxiety and become a barrier to participation. This could be mitigated by the development of concise videos over time versus being given all information on a single day in clinic before surgery. One clinician provided a method to condense information into smaller fragments that engage patients.

I think that the biggest thing is and it sounds like, a principle fact in education is in general to have like one or two take home messages per video, so there would be more value to having many smaller videos that have a tidbit of information, compared to a longer video that has too much information.

(Clinician 8)

Lastly, an important barrier that emerged from the findings was around tracking adherence to the program. Tracking adherence to the program allows us to identify which patients are completing the online material, how many times they maybe be reviewing the material and which ones are struggling with the material or are inconsistent/forgetting to do it.

So having some interactive things throughout might help to track adherence to the program to and check in to see what is really being absorbed by the patients and what's missing…. having a calendar as well for the exercise tracker seems to be helping so you could simply ask them to check off which days that they watched the videos.

(Clinician 8)

## 4 Discussion

Traditional health education programs that have been created by clinicians with minimum patient consultation can result in the poor patient engagement and adherence. Our study has qualitatively explored patients' and clinicians' preferences and follows the traditional principles of patient co‐creation. These principles will lead to a preoperative education program that can facilitate engagement and adherence. Patient and clinician preferences differed, and we were able to summarize suggestions on access, content, platforms, and factors that would facilitate the development of an accessible program for both groups. The qualitative findings from this study parallel our previous quantitative work and provide further depth to both patient and clinician's suggestions [16]. Both studies support the creation of a program that can be more useful to patients and clinicians. Future work is required to design a preoperative program and evaluate its benefits in a population of patients awaiting total shoulder replacement.

Health decision‐making is influenced by health information delivery and retrieval. Patients commonly access health information directly from healthcare providers, through internet searches and from other patients who have already received similar treatment. While some patients are fortunate to know other people who have had a similar surgery, not every patient might. As shown in previous literature, having healthcare professionals design preoperative education programs that are accessible to patients before surgery, can standardize the type of information patients are receiving [17]. From previous suggestions based on published preoperative programs, researchers can include a section in the education program that features previous patients who have had a shoulder replacement and patient opinions on how to be successful for this procedure. Inputting a section that is informed by patient stories can allow for a more personable and individualized program that reinforces the process of patient codesign.

The patient codesign process also balances content that meets both patient and clinician needs. From the various healthcare providers and patients, we have been able to narrow down key components of content that should be included within a preoperative program. Having several perspectives allowed us to understand what content is important to include, how much of that content to include and which content did not need to be emphasized in a program. In contrast to our quantitative work, exploring the rationale behind participant choices helped us to further inform the content of our program. From our data, topics that were often mentioned by our participants to include will be featured as independent modules in the program, ie. exercises before and after surgery, recovery timeline, what to expect on the day of surgery etc. Topics that were less frequently mentioned will still be included in the program, but as additional reading material or web links to view if a participant requires more information on that topic, ie. information about driving, pathology, or specifics of the implant itself. Incorporating the patient perspective allows us to better understand how to potentially improve engagement and adherence to the program. Literature shows that having a more patient‐centered approach to designing health education, allows patients to feel heard as valued member on the team. Additionally, including a variety of healthcare workers rather than only focusing on surgeons as the primary resource, can allow for a more interdisciplinary approach to creating a preoperative program. Each healthcare member has a unique role and perspective in the care of patients undergoing shoulder replacement.

Lastly when designing a patient education program, deciding on the appropriate platform is critical to ensure accessibility. As shown in our study, a multimedia program of virtual education and a physical workbook of the program with the same information as an online program can be one method to increase accessibility to all users with a variety of learning styles.

During design phases, researchers should address any assumptions or psychological barriers that may be present when working with older adults and technology. Many older adults face issues with dexterity, fine motor control, visual acuity, and audio acuity, which may hinder their ability to use smaller devices like a cellphone, but they are willing to participate in a program that is featured on a webpage and is computer or tablet‐friendly. Although not all patients have access to smartphones or tablets, the use of mobile technology is increasing annually. The overwhelming majority of Canadians aged 15 to 44 (96%) reported having a smartphone in 2020, as did most Canadians aged 45 to 64 (87%). Seniors who reported owning a smartphone rose from 43% in 2018% to 54% in 2020 [18]. Therefore, as researchers design programs, understanding these aspects of design implementation within their population can strengthen adherence to the overall program.

Lastly, health literacy and patient education intersect when designing preoperative programs. Health literacy is an individual's knowledge, motivation, and competency to access, understand, appraise, and apply health information to make judgements in everyday life concerning health care. Several studies have shown that patient education material can be more understandable when summarized by importance, presented using an active voice using small phrases and simpler terms, avoiding medical jargon, and adding descriptive pictures. As shown in a study by the National Assessment of Adult Literacy, 12% of adults have proficient health literacy, yet 81% of the patient education materials provided by the American Academy of Orthopedic Surgeons had a readability score above the 8th‐grade level [19]. These findings align with the concerns raised by our patients who expressed the importance of using simple terms to explain medical terminology. Researchers need to ensure that the readability of preoperative education material is correctly aligned with the overall health literacy of orthopedic patients.

When comparing the results from this study to our previous quantitative work [16], there were many similarities present between the ways participants ranked methods of accessing information, education content and device choices. While this study was a subset of the patient population from our previous quantitative work, we were able to further provide more in‐depth analyses of our participants choices. Using traditional patient codesign principles is important to incorporate early in the design phase rather than later. As shown in previous patient codesign programs, early incorporation of relevant key groups ensures a more efficient design process with fewer iterations of the program. This results in less time and costs for the research team and hospital [7, 20].

Overall, our study had both limitations and strengths. Firstly, we recruited only English‐speaking patients who were predominately from Ontario, Canada. However, our clinicians were from all over Canada. Future studies might want to focus on non‐English speaking participants with a variety of socioeconomic status to gain a wider experience. However, our sample did provide rich information about the experiences of our patients and health providers, with the goal of providing insight using the traditional principles of patient codesign. The core concepts from our research should be transferable to other centers wanting to develop similar patient‐centered education materials.

## 5 Conclusion

In summary, a multimodal program of both a website with videos and a written booklet, that covers basic information regarding the surgery, timelines for recovery, immobilization/sling use, use of therapeutic devices/aids post‐surgery, patient expectations to improve surgery satisfaction, postoperative restrictions, pain management, rehabilitation after surgery, home supports and the use of caregivers is desired by both patients and clinicians.

## Conflicts of Interest

The authors declare no conflicts of interest.

## Data Availability

The data that support the findings of this study are available on request from the corresponding author. The data are not publicly available due to privacy or ethical restrictions.

## References

[1] M. F. Lemos , S. V. Lemos‐Neto , L. Barrucand , N. Verçosa , and E. Tibirica , “Preoperative Education Reduces Preoperative Anxiety in Cancer Patients Undergoing Surgery: Usefulness of the Self‐Reported Beck Anxiety Inventory,” Brazilian Journal of Anesthesiology (English Edition) 69 (2019): 1–6. 10.1016/j.bjane.2018.07.004 PMC9391836 30401475

[2] R. Furtado , J. C. MacDermid , C. Ziebart , D. Bryant , and K. J. Faber , “Preoperative Patient Education Programs for Orthopaedic Surgery: What Do the Programs Include? How Are They Delivered? What Are the Knowledge Gaps? A Scoping Review of 46 Studies,” Journal of Orthopaedic and Sports Physical Therapy 52, no. 9 (2022): 572–585. 35802819 10.2519/jospt.2022.10614

[3] D. Aydin , J. Klit , S. Jacobsen , A. Troelsen , and H. Husted , “No Major Effects of Preoperative Education in Patients Undergoing Hip or Knee Replacement—A Systematic Review,” Danish medical journal 62, no. 7 (2015): A5106. 26183051

[4] K. Johansson , S. Salanterä , and J. Katajisto , “Empowering Orthopaedic Patients Through Preadmission Education: Results From a Clinical Study,” Patient Education and Counseling 66 (2007): 84–91, 10.1016/j.pec.2006.10.011. 17161934

[5] A. Louw , I. Diener , D. S. Butler , and E. J. Puentedura , “Preoperative Education Addressing Postoperative Pain in Total Joint Arthroplasty: Review of Content and Educational Delivery Methods,” Physiotherapy Theory and Practice 29 (2013): 175–194, 10.3109/09593985.2012.727527. 23035767

[6] G. Kok , N. H. Gottlieb , G. J. Y. Peters , et al., “A Taxonomy of Behaviour Change Methods: An Intervention Mapping Approach,” Health Psychology Review 10 (2016): 297–312, 10.1080/17437199.2015.1077155. 26262912 PMC4975080

[7] D. Kennedy , A. Wainwright , L. Pereira , et al., “A Qualitative Study of Patient Education Needs for Hip and Knee Replacement,” BMC Musculoskeletal Disorders 18 (2017): 413, 10.1186/s12891-017-1769-9. 29025397 PMC5639777

[8] T. L. McCarron , T. Noseworthy , K. Moffat , et al., “Understanding the Motivations of Patients: A Co‐Designed Project to Understand the Factors Behind Patient Engagement,” Health Expectations 22, no. 4 (2019): 709–720. 31379094 10.1111/hex.12942 PMC6737762

[9] L. M. Maher , B. Hayward , P. Hayward , and C. Walsh , “Increasing Patient Engagement in Healthcare Service Design: A Qualitative Evaluation of a Co‐Design Programme in New Zealand,” Patient Experience Journal 4, no. 1 (2017): 23–32.

[10] L. R. Knudsen , K. Lomborg , and A. de Thurah , “Design and Development of an E‐Learning Patient Education Program for Self‐Management Support in Patients With Rheumatoid Arthritis,” PEC Innovation 1 (2022): 100004. 37364010 10.1016/j.pecinn.2021.100004 PMC10194095

[11] P. A. Kelly and P. Haidet , “Physician Overestimation of Patient Literacy: A Potential Source of Health Care Disparities,” Patient Education and Counseling 66, no. 1 (2007): 119–122. 17140758 10.1016/j.pec.2006.10.007

[12] E. R. Wagner , K. X. Farley , I. Higgins , J. M. Wilson , C. A. Daly , and M. B. Gottschalk , “The Incidence of Shoulder Arthroplasty: Rise and Future Projections Compared With Hip and Knee Arthroplasty,” Journal of Shoulder and Elbow Surgery 29, no. 12 (2020 Dec): 2601–2609, Epub 2020 Jun 9. PMID: 33190759, 10.1016/j.jse.2020.03.049. 33190759

[13] S. Thorne , Interpretive Description: Qualitative Research for Applied Practice (Routledge, 2016).

[14] S. Thorne , S. R. Kirkham , and J. Macdonald‐Emes , “Interpretive Description: A Noncategorical Qualitative Alternative for Developing Nursing Knowledge,” Research in Nursing & Health 20, no. 2 (1997): 169–177. 9100747 10.1002/(sici)1098-240x(199704)20:2<169::aid-nur9>3.0.co;2-i

[15] R. Furtado , J. C. MacDermid , D. M. Bryant , K. J. Faber , and G. S. Athwal , “Interpretation and Content Validity of the Items of the Numeric Rating Version Short‐WORC to Evaluate Outcomes in Management of Rotator Cuff Pathology: A Cognitive Interview Approach,” Health and Quality of Life Outcomes 18, no. 1 (2020): 88. 32228622 10.1186/s12955-020-01339-7 PMC7106799

[16] R. Furtado , J. C. MacDermid , D. Bryant , K. J. Faber , D. S. Drosdowech , and G. S. Athwal , “Balancing Clinician and Patient Priorities for Total Shoulder Replacement Preoperative Education Programs,” Patient Education and Counseling 112 (2023): 107759. 37075651 10.1016/j.pec.2023.107759

[17] L. Gottlieb , R. Tobey , J. Cantor , D. Hessler , and N. E. Adler , “Integrating Social and Medical Data to Improve Population Health: Opportunities and Barriers,” Health Affairs 35, no. 11 (2016): 2116–2123. 27834254 10.1377/hlthaff.2016.0723

[18] S. C. Government of Canada , *So Long Landline, Hello Smartphone*, (2023), https://www.statcan.gc.ca/o1/en/plus/3582-so-long-landline-hello-smartphone#shr-pg-pnl1.

[19] A. Eltorai , S. Ghanian , C. Adams Jr. , C. Born , and A. Daniels , “Readability of Patient Education Materials on the American Association for Surgery of Trauma Website,” Archives of Trauma Research 3, no. 2 (2014): e18161, 10.5812/atr.18161. 25147778 PMC4139691

[20] P. K. Edwards , S. C. Mears , and C. Lowry Barnes , “Preoperative Education for Hip and Knee Replacement: Never Stop Learning,” Current Reviews in Musculoskeletal Medicine 10 (2017): 356–364, 10.1007/s12178-017-9417-4. 28647838 PMC5577053

